# Supplementary material for: Application of bubble streams to control biofouling on marine infrastructure—pontoon-scale implementation
Source: PeerJ. 2023 Sep 7;11:e16004. doi: 10.7717/peerj.16004 (PMC10493092; doi:10.7717/peerj.16004)
Supplement: Supplemental Information 1 — σ2 indicates the model residual variance. τ00 indicates the random effects variance component of Block. Significant values are indicated in bold. n = 197. [file peerj-11-16004-s001.docx]

Table S1. Results of generalised linear mixed models examining the fixed effects of treatment and months on the percentage cover of bare space, biofilm and macrofouling. σ2 indicates the model residual variance. τ00 indicates the random effects variance component of Block. Significant values are indicated in bold. n = 197

|  | **Bare space** | | | **Macrofouling** | | | **Biofilm** | | |
| --- | --- | --- | --- | --- | --- | --- | --- | --- | --- |
| *Predictors* | *Estimates* | *CI* | *p* | *Estimates* | *CI* | *p* | *Estimates* | *CI* | *p* |
| (Intercept) | 11.39 | 8.27 – 15.67 | **<0.001** | 0.13 | 0.08 – 0.20 | **<0.001** | 0.04 | 0.02 – 0.07 | **<0.001** |
| time month [1] | 0.52 | 0.35 – 0.79 | **0.002** | 1.63 | 0.95 – 2.80 | 0.076 | 1.06 | 0.57 – 1.97 | 0.857 |
| time month [2] | 0.35 | 0.24 – 0.53 | **<0.001** | 2.04 | 1.20 – 3.47 | **0.008** | 1.51 | 0.81 – 2.79 | 0.191 |
| time month [3] | 0.00 | 0.00 – 0.00 | **<0.001** | 23.45 | 13.69 – 40.19 | **<0.001** | 8.45 | 4.74 – 15.05 | **<0.001** |
| time month [4] | 0.00 | 0.00 – 0.00 | **<0.001** | 22.87 | 13.44 – 38.90 | **<0.001** | 8.75 | 4.91 – 15.58 | **<0.001** |
| treatment [Control] | 1.17 | 0.74 – 1.84 | 0.500 | 0.86 | 0.49 – 1.52 | 0.614 | 1.00 | 0.54 – 1.86 | 1.000 |
| time month [1] * treatment [Control] | 0.59 | 0.33 – 1.06 | 0.076 | 1.41 | 0.65 – 3.06 | 0.378 | 1.27 | 0.52 – 3.07 | 0.601 |
| time month [2] * treatment [Control] | 0.01 | 0.01 – 0.02 | **<0.001** | 0.17 | 0.08 – 0.38 | **<0.001** | 198.88 | 79.25 – 499.09 | **<0.001** |
| time month [3] * treatment [Control] | 0.78 | 0.37 – 1.64 | 0.512 | 1.51 | 0.72 – 3.19 | 0.278 | 0.78 | 0.35 – 1.73 | 0.535 |
| time month [4] * treatment [Control] | 0.86 | 0.41 – 1.80 | 0.681 | 0.12 | 0.06 – 0.25 | **<0.001** | 9.46 | 4.25 – 21.03 | **<0.001** |
| **Random Effects** | | | | | | | | | |
| σ^2^ | 0.00 | | | 0.00 | | | 0.00 | | |
| τ_00_ | 0.00 _block_ | | | 0.03 _block_ | | | 0.02 _block_ | | |
| ICC |  | | | 1.00 | | | 1.00 | | |
| N | 2 _block_ | | | 2 _block_ | | | 2 _block_ | | |
| Observations | 197 | | | 197 | | | 197 | | |
| Marginal R^2^ / Conditional R^2^ | 1.000 / NA | | | 0.989 / 1.000 | | | 0.995 / 1.000 | | |
